# High Prevalence of *Schistosoma japonicum* Infection in Carabao from Samar Province, the Philippines: Implications for Transmission and Control

**DOI:** 10.1371/journal.pntd.0001778

**Published:** 2012-09-20

**Authors:** Catherine A. Gordon, Luz P. Acosta, Darren J. Gray, Remigo M. Olveda, Blanca Jarilla, Geoffrey N. Gobert, Allen G. Ross, Donald P. McManus

**Affiliations:** 1 Molecular Parasitology Laboratory, Infectious Diseases Division, Queensland Institute of Medical Research, Brisbane, Australia; 2 Infectious Disease Epidemiology Unit, School of Population Health, University of Queensland, Brisbane, Australia; 3 Department of Immunology, Research Institute of Tropical Medicine, Manila, Philippines; 4 Griffith Health Institute, Griffith University, Queensland, Australia; University of Melbourne, Australia

## Abstract

*Schistosoma japonicum* is endemic in the Philippines, China and Indonesia, and infects more than 40 mammalian host species, all of which can act as reservoirs of infection. In China, water buffaloes have been shown to be major reservoirs of human infection. However, in the Philippines, carabao have not been considered important reservoir hosts for *S. japonicum* due to the low prevalence and infection intensities reported, the only exception being a qPCR-based study indicating 51% of carabao were *S. japonicum*-positive. However, the low prevalence found for the same animals when using conventional copro-parasitological techniques means that there is still confusion about the role of carabao in the transmission of schistosomiasis japonicum. To address this inconsistency, and to shed light on the potential role of carabao in the transmission of *S. japonicum* in the Philippines, we undertook a pilot survey, collecting fecal samples from animals in Western Samar Province and we used a combination of molecular and copro-parasitological techniques to determine the prevalence and intensity of *S. japonicum*. We found a high prevalence of *S. japonicum* in the carabao using a validated real-time PCR (qPCR) and a copro-parasitological tool, the formalin-ethyl acetate sedimentation (FEA-SD) technique. A much lower prevalence of *S. japonicum* was recorded for the same fecal samples using conventional PCR, the Kato-Katz technique and miracidial hatching. These results suggest that, due to their low diagnostic sensitivity, traditional copro-parasitological techniques underestimate infection in carabao. The use of FEA-SD and qPCR provides a more accurate diagnosis. Based on these findings, the role of bovines in the transmission of *S. japonicum* appears to be more important in the Philippines than previously recognized, and this may have significant implications for the future control of schistosomiasis there, particularly as, in contrast with previous surveys, we found an unprecedented high prevalence of *S. japonicum* in humans.

## Author Summary

Schistosomiasis japonica is endemic in China, the Philippines and parts of Indonesia. Extensive research efforts have shown that water buffaloes are major reservoir hosts for transmission of *Schistosoma japonicum* in China. In contrast, comparatively fewer studies have been undertaken on the reservoir hosts for schistosomiasis japonica in the Philippines, although several reports have indicated that water buffaloes (carabao) are unimportant in transmission. Here, we undertook a pilot survey, using a new diagnostic tool (formalin-ethyl acetate sedimentation; FEASD), combined with real-time PCR (qPCR) for the detection of *S. japonicum* eggs in fecal samples, and report high prevalence of *S. japonicum* in carabao from Samar province. This study suggests that water buffaloes in the Philippines, as in China, are major reservoir hosts for the transmission of schistosomiasis. Future integrated control, including interventions targeting bovines, would likely reduce the prevalence of infection in humans.

## Introduction

*Schistosoma japonicum* is the causative agent of schistosomiasis in China, the Philippines and Indonesia. In the Philippines, 10 of 16 regions (administrative divisions) have reported cases of clinical schistosomiasis, with 6.7 million people living in the endemic areas [1]–[3]. Furthermore, within these endemic areas, 1.8 million people are considered to be directly exposed to potential infection through their daily lifestyle and contact with water including farming, fishing, domestic activities (bathing and washing) and recreation [1], [2]. Currently, there are 560,000 known cases of schistosomiasis in the Philippines [4].

Unlike African schistosomiasis, schistosomiasis japonica is a zoonosis, naturally infecting more than 40 mammalian host species [5]–[7]. Large mammals, such as bovines, excrete substantial amounts of feces and, therefore, have a high contamination index. Daily fecal excretion rates have been estimated to be as high as 25 kg for cattle and ≥50–60 kg for water buffaloes (*Bubalis bubalis*) [8]. In China, a number of drug intervention trials have demonstrated the importance of bovines in the transmission of *S. japonicum* to humans [8]–[12]. As a result, water buffaloes in China are well recognized as the major reservoir hosts for schistosomiasis in the lake and marshland areas, contributing up to 75% of subsequent transmission to humans [8]–[12].

In contrast to China, limited research has been undertaken in the Philippines to determine whether water buffaloes (carabao; *Bubalus bubalis carabanesis*) act similarly as major reservoir hosts. The majority of information comes from studies undertaken more than 10 years ago, which suggested instead a central role for rodents in *S. japonicum* transmission [13]–[16]. Additionally, dogs and cats have also been implicated as potentially important hosts, while, based on low prevalence data, carabao have been considered as less important [5], [7], [17], [18]. For example, on Samar, the easternmost island in the Visayas Group, previous studies using copro-parasitological techniques have demonstrated very low prevalence (3.8%) of *S. japonicum* infection in carabao using the Kato-Katz method (KK) [18]. In direct contrast, a more recent study has shown that 51.5% of carabao on the nearby island of Leyte were test-positive for *S. japonicum* using real-time PCR (qPCR), although few or no eggs/hatched miracidia were found using the KK, the Miracidial Hatching Technique (MHT) and the Danish Laboratory Bilharziasis (DBL) technique [19]. Despite these inconsistencies, the investigators of this study suggested that the actual prevalence of *S. japonicum* infection in carabao may be much higher than previously considered, and that the true role and impact that these carabao have on schistosome transmission in the Philippines should be re-evaluated [19].

Accordingly, in this pilot study, we have used a combination of qPCR, conventional PCR (PCR), modified KK, MHT and a novel copro-parasitological tool, the formalin-ethyl acetate sedimentation (FEA-SD) technique, recently developed in China for improving the detection of *S. japonicum* eggs in the feces from bovines and other ruminant hosts (Gordon *et al.*, 2012, accompanying paper). We report a high prevalence of *S. japonicum* in carabao and humans on the island of Samar, based on both the presence of fecal eggs and the quantification of *S. japonicum* DNA in fecal samples. We discuss the implications of these results in terms of the transmission of schistosomiasis japonica and its future control in the Philippines.

## Materials and Methods

### Ethics Statement

Informed written consent was received from all human participants in the study and ethical approval for human work given from Ethics Committee of the Research Institute of Tropical Medicine (RITM), Manila, and the Queensland Institute of Medical Research (QIMR) Human and Animal Research Ethics Committees (Project Number P288). The conducts and procedures involving animal experiments were approved by the Animals Ethics Committee of the Queensland Institute of Medical Research (Project Number P288) and the Ethics Committee of the Research Institute of Tropical Medicine (RITM), Manila. Informed written consent was received from all animal owners to collect samples. This study was performed in accordance with the recommendations of the Australian code of practice for the care and use of animals for scientific purposes, 2004.

### Study design

We carried out a cross-sectional survey in Samar Province, the Philippines, to determine the *S. japonicum* infection status in carabao and humans using a range of diagnostic techniques. This study took place in September 2010. Primary endpoints were carabao and human prevalence and intensity of infection; secondary end points were sensitivity and specificity of the diagnostic techniques employed.

### Study area

The study was undertaken in three barangays (villages); Cantaguic, Diaz and Hinugacuan and one dairy farm, near Hinugacuan, all in Samar Province. The barangays had previously been part of an earlier survey in 2003 [5], [17], [18], [20], after which praziquantel-treatment of infected humans was carried out. In the intervening years, clinical mass treatment was also performed in these barangays, although details of the treatment coverage were not recorded. As far as we are aware, there has been no treatment of bovines or other animals in these locations. Bovines from the four locations were sampled for fecal examination. Stool samples were collected from 44 bovines. We also collected 52 human fecal samples from Cantaguic barangay.

### Study procedures

Carabao fecal samples were collected either rectally by trained veterinary personnel or from freshly deposited stools collected from individual animals by the carabao owners. Two separate stool samples were sought from all carabao on different days. For human samples, individuals were given stool cups which were collected over a period of four days. Two stool samples on separate days were sought from all participants.

### Copro-parasitology techniques

All carabao fecal samples were subjected to analysis using a modified KK, the formalin-ethyl acetate sedimentation method (FEA-SD), and PCR and qPCR techniques. Additionally, the MHT was performed on 21 stool samples from 19 individual carabao. All human samples were subjected to testing using KK, PCR and qPCR arrays.

#### Modified Kato-Katz (KK)

A modified KK was performed on all collected bovine stool samples, according to a published protocol [21]. Briefly, the collected stool sample was pressed through gauze and the non-retained material used to fill a standard volume template representing approximately 50 mg of fecal material. This material was then transferred to a glass slide where cellophane soaked in glycerin was pressed against a firm surface to spread the stool evenly which was then viewed under a microscope. Two slides per stool sample were read blind and independently by four microscopists.

#### Miracidial hatching test (MHT)

The MHT was performed as previously described [22] with minor modifications. Briefly, ∼50 g of bovine stool were homogenized and sieved. The sediment was collected, placed into a triangular flask filled with fresh, unchlorinated water (pH 7.8) and left in a well-lit room at 24–30°C. The neck of the flask was illuminated with a lamp and examined after 4, 8, 12 and 24 hours to detect the presence of hatched miracidia.

#### Formalin-ethyl acetate sedimentation technique (FEA-SD)

The recently developed FEA-SD method (Gordon et al., accompanying paper) was used with some minor modifications. Briefly, 50 g of bovine stool were sieved sequentially through a 40–60 mesh (Tyler scale) (234–380 µm (pore opening size)) sieve and then a 260 mesh (61 µm) sieve. The sediment was washed into a 50 ml falcon tube and mixed by shaking and sedimented in the tube by leaving for 30 min. The supernatant was removed and the sediment re-suspended in 10% (v/v) formalin and natural sedimentation repeated twice more. After the final sedimentation step, the 50 ml tube was topped up to 50 ml and vortexed. Ten ml of this final suspension was transferred to two 15 ml tube (5 ml suspension per tube), centrifuged for 10 min at 1800× g, the supernatant removed, and the pellet re-suspended in 7 ml of 10% (v/v) formalin and 2 ml of 100% ethyl acetate (v/v), and then centrifuged at 500× g for 10 min. This resulted in a four layer sedimentation, of which the top three layers were removed. Water was added to each tube to adjust the volume up to a minimum of 5 ml and an equal volume of 10% (w/v) potassium hydroxide solution was added. After vortexing, the tubes were left overnight at 37°C. The next day, the suspension was centrifuged at 1800× g for 10 min, the resultant pellet was washed once with water and then re-suspended in 6 ml of 10% (v/v) formalin solution. The solution was examined under a microscope following the pipetting of 200 µl of the suspension on to a glass slide. Two ×200 µl aliquots were examined, and eggs counted by each of four independent microscopists (1.6 ml suspension in total).

### Molecular analysis

#### DNA extraction from fecal samples

Genomic DNA was isolated from bovine and human fecal samples using QIAamp mini stool mini kits (QIAGEN, Hilden, Germany). DNA concentration was determined using a NanoDrop 2000 (Thermo Scientific) and all samples were diluted to 50 ng/µl for analysis.

#### Conventional polymerase chain reaction (PCR)

The primers used for the PCR were employed in a previously reported assay [23], which amplifies fragments of the cytochrome oxidase 2 (*cox2*) and NADH dehydrogenase 6 (*nad6*) mitochondrial genes, and are specific for *S. japonicum*. A reaction mix of 18 µl was prepared containing 11.25 µl H_2_O, 2 µl kit provided buffer, 1.0 mM MgCl_2_, 800 µM deoxyribonucleotide triphosphate (dNTPs), 1.25 units/reaction Gold AmpliTaq (Applied Biosystems, Foster City, CA) and 5 µM of each primer. To this mix, 2 µl isolated template DNA (50 ng/µl) were added in a total reaction volume of 20 µl. The PCR was performed on a thermocycler (Mastercycler, Eppendorf) under previously described conditions [23], and PCR products were resolved by electrophoresis on 2% (w/v) agarose gels and stained with ethidium bromide.

#### Real-time PCR (qPCR)

The primers employed were of a previously reported [19], [24] PCR assay which amplifies a fragment of the NADH dehydrogenase I (*nad1*) mitochondrial gene. Reaction mixtures of 22 µl were prepared containing 12.2 µl SYBR Green (Invitrogen), 5 µM of each primer and 7.8 µl of H_2_O. To this, 2 µl of DNA (50 ng/µl) template was added for a total reaction volume of 24 µl. The PCR conditions were as follows: 50°C initialization for 2 min, 95°C denaturation for 10 min, followed by 45 cycles of denaturation at 95°C for 15 sec, annealing at 60°C for 60 sec, extension at 72°C for 90 sec and a final dissociation phase at 60–95°C. The PCR was performed using a conventional thermocycler standard (Corbett RotorGene 6000). The results were quantified for in relation to eggs per gram [EPG] (1–120 eggs per gram) using Ct (cycle threshold) scores. Melt curve analysis was performed after each qPCR. To determine the equivalent egg number relating to Ct scores, a series of seeding and dilution experiments were performed. For seeding experiments, stool samples known to be negative for *S. japonicum* were seeded with a known number of eggs and DNA extracted from these seeded samples. A standard curve using the dilutions and the results of the seeding experiments was then created to determine a range of Ct scores which corresponded to a known number of eggs. This curve was then used to compare the unknown samples from the study and to determine egg numbers.

Amplicons for both cPCR and qPCR were sequenced, thereby confirming each DNA target sequence.

### Data management and data analyses

Microsoft Excel and SAS software (SAS Institute, Cary, NC) were used for data analyses. Diagnostic sensitivity and specificity were calculated using the following formulae:
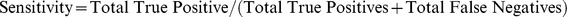


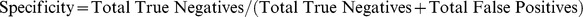
The bovine contamination index (BCI) was derived using the published formula [25]:
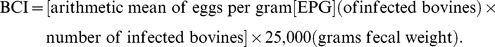
A conservative approach was taken when deriving the BCI by using 25 kg of feces excreted per day in our calculations; thus the value 25, 000 was used. It has been shown in China that bovines can excrete 25–50 kg of bovine feces per day.

## Results

### Molecular analysis (qPCR)

The seeding experiments showed that the qPCR was sufficiently sensitive to detect a single *S. japonicum* egg. Dilution experiments showed that the qPCR could detect as little as 14 pg (approximately 30% of the DNA in one egg). This value was obtained by extracting DNA from a known number of eggs and calculating for the content of one egg.

### Prevalence

The prevalence of *S. japonicum* infection in carabao was high (95.45%; 95% CI 89.05–100%) when the results achieved using all diagnostic techniques used were combined (Table 1). The prevalence of *S. japonicum* infection obtained from the FEA-SD and qPCR techniques were 93.18% (95% CI 85.43–100%) and 90.91% (95% CI 82.07–99.75%), respectively, and proved to be the most sensitive for detecting *S. japonicum* in carabao, whereas the prevalence estimated based on the use of KK, MHT and conventional PCR were 25% (95% CI 11.68–38.32%), 19.05% (95% CI 0.86–41.24%) and 31.82% (95% CI 17.49–46.14), respectively. MHT was performed on 21 samples from 19 carabao (Table 1). Prevalence was also calculated by barangay using FEA-SD and qPCR data (Table 2).

**Table 1:** Prevalence and infection intensity of *S. japonicum* in carabao determined using different diagnostic procedures.

	N	Number positive	Prevalence % (CI**)	GMEPG^1^ (CI**)
**Total examined**	44	42	95.45 (89.05–100)	N/A
**Diagnostic technique**				
FEA-SD	44	41	93.18 (85.43–100)	1.17 (0.88–1.57)
qPCR	44	40	90.91 (82.07–99.75)	6.14 (4.55–8.27)
PCR	44	14	31.82 (17.49–46.14)	N/A
KK	44	11	25 (11.68–38.32)	4.69 (2.81–7.84)
MHT*	19	4	19.05 (0.86–41.24)	N/A

1 Geometric mean eggs per gram (GMEPG).

* MHT performed on 21 samples from 19 bovines.

** 95% CI.

**Table 2:** Prevalence and infection intensity of *S. japonicum* in carabao by barangay/dairy farm using the FEA-SD data.

Barangay	N	No. Positive	FEA-SD Prevalence % (CI*)	FEA-SD GMEPG^1^ (CI*)	qPCR Prevalence % (CI*)	qPCR GMEPG^1^ (CI*)
Hinugacuan	8	7	87.5 (57.94–100)	0.55 (0.23–1.35)	87.5 (57.94–100)	7.93 (1.90–33.19)
Diaz	7	7	85.71 (50.76–100)	0.63 (0.21–1.91)	100 (N/A)	5.8 (2.93–11.40)
Cantaguic	7	6	85.71 (50.76–100)	1.06 (0.54–2.09)	71.43 (26.3–100)	4.35 (2.96–6.40)
Dairy farm	22	22	100 (N/A)	1.82 (1.34–2.48)	95.45 (86–100)	6.24 (4.21–9.24)

1 Geometric mean eggs per gram (GMEPG).

* 95% CI.

The prevalence of *S. japonicum* in humans varied depending on the technique used, with the highest prevalence, 92.31% (95% CI 84.82–99.8%), estimated using qPCR and the lowest (30.77%, (95% CI 17.79–43.74%)) using KK (Table 3). A prevalence of 75% (95% CI 62.83–87.17%) was detected by conventional PCR (Table 3). Human samples test-positive by qPCR, but negative by KK, were examined again by microscopy, after sieving, sedimenting and KOH digestion of each sample. *S. japonicum* eggs were found in all of the samples examined by one of the authors (CAG).

**Table 3:** Prevalence and infection intensity of *S. japonicum* in humans determined using different diagnostic procedures.

	N	Number positive	Prevalence % (CI**)	GMEPG^1^ (CI**)
**Total examined**	52	50		
**Diagnostic technique**				
qPCR		50	92.31% (84.82–99.8)	16.54 (10.06–27.18)
PCR		39	75% (62.83–87.17)	N/A
KK		16	30.77%, (17.79–43.74)	4.90 (2.48–9.67)

1 Geometric mean eggs per gram (GMEPG).

** 95% CI.

### Intensity

The intensity of *S. japonicum* infection in carabao (geometric mean eggs per gram (GMEPG) feces) was determined using the modified KK, FEA-SD and qPCR methods (Table 1). The GMEPG obtained by qPCR was calculated from the average and standard deviation of Ct scores compared with a standard curve prepared using a DNA dilution series. Analysis of the FEA-SD results gave lower overall GMEPG than the modified KK and qPCR for carabao (Table 1).

The intensity of *S. japonicum* infection by age, sex and individual barangay and the dairy farm for each carabao was also calculated using the FEA-SD and qPCR data (Table 2). The intensity of infection by FEA-SD was highest in carabao from the dairy farm (1.82), with the lowest values in Diaz (0.63) and Hinugacuan (0.55) (Table 2). Infection intensity did not vary with the age or sex of the carabao (data not shown).

For the samples from humans, a GMEPG of 16.54 (95% CI 10.06–27.18) was found with the qPCR and 4.90 (95% CI 2.48–9.67) with the KK technique; no differences in intensity of infection according to age or sex were recorded.

### Sensitivity and specificity of the copro-parasitological and molecular techniques

Sensitivity and specificity were calculated for each of the techniques used, with a combination of any positive results from all techniques used as the reference value. For carabao, qPCR and FEA-SD provided the highest sensitivities of 95.25% (95% CI 88.82–100) and 97.62% (95% CI 93.01–100), respectively, whereas the conventional PCR (33.33%; 95% CI 19.07–47.59), KK (26.19%; 95% CI 12.89–39.49) and MHT (21.05%; 95% CI 2.72–39.38; based on the test results for 19 fecal samples) had lower sensitivity. All techniques had a specificity of 100%.

With the samples from humans, qPCR was the most sensitive (94.0%; 95% CI 87.42–100), followed by PCR (78.0%; 95% CI 66.52–89.48) and KK (32.0%; 95% CI 19.07–44.93).

### Bovine contamination index (BCI)

We used 25 kg as a conservative estimate for the total daily fecal output for carabao, compared with 25–50 kg reported in the literature for Chinese water buffaloes [8]. Our calculations indicated each carabao excreted, on average, 41,750 *S. japonicum* eggs per day into the environment (Table 4).

**Table 4:** Bovine contamination index (BCI)* for carabao in Samar calculated using the arithmetic mean EPG of the FEA-SD data.

Barangay	Arithmetic mean EPG	Number infected	BCI overall	BCI per carabao
Cantaguic	1.25	6	187500.00	31250.00
Hinugacuan	0.80	7	140000.00	20000.00
Diaz	0.97	6	145500.00	24250.00
Dairy farm	2.25	22	1237500.00	56250.00
All	1.67	41	1711750.00	41750.00

* Calculated using 25 kg as the daily fecal output for a carabao.

## Discussion

In this pilot study, we show that the prevalence of *S. japonicum* infection in carabao in Samar is substantially higher than reported previously in this and other regions of the Philippines. A recent study on the island of Leyte reported a prevalence of 52% in carabao by qPCR [19], whereas the results obtained by the same group using the MHT, KK and DBL techniques on carabao from Samar detected less than 4% prevalence [19]. A previous study from Leyte using the merthiolate iodine formalin concentration technique (MIFC) and circumoval precipitin test (COPT) found a combined prevalence of 9.8% in carabao [26], whereas another survey of 50 Samar barangays [5] revealed very low prevalence (2.1%) in carabao using the DBL (Danish Bilharzias Laboratory) technique. Surprisingly, previous studies of the same three barangays that we surveyed in Samar indicated that all carabao present were test-negative for *S. japonicum* infection [5], [17], [18], [20]. This contrasts to our findings showing that the majority of carabao in Cantaguic, Diaz, Hinugacuan, and the dairy farm (near Hinugacuan) were infected with *S. japonicum*, including 100% of carabao at Diaz and the dairy farm, resulting in an overall prevalence of 95.45%. This high prevalence was obtained due to the diagnostic sensitivity of the FEA-SD and qPCR techniques employed, with the traditional KK technique being far less sensitive.

The sensitivity of each of the diagnostic methods used in this study varied substantially, with the KK technique and MHT proving to be the least sensitive. It is recognized that the sensitivity of the KK increases with the number of stool samples taken and the number of slides examined per sample [27], [28]. In the present study, two stool samples and two slides per sample were assessed, such that it is likely that increasing the number of slides examined would have improved the sensitivity of the method. Additionally, we were unable to effectively control the temperature or water pH for the MHT under the prevailing field conditions in Samar, both of which are critical for optimal performance of the test.

The use of qPCR is likely to result in a less accurate egg count than physical counting of eggs, as only approximations can be made with the technique after using a standard curve of known DNA concentrations (which correspond to a known number of eggs). Additionally for the qPCR method, only ∼200 µg of stool were used for the analysis, which is much lower than that for the FEA-SD which uses a final volume equivalent to 10 g of stool. The modified KK showed a higher intensity of infection than the FEA-SD due to the lack of sensitivity of the KK. The KK usually detects only high intensity infections which may represent only a small proportion of the total animals infected [28]–[32]. The GMEPG is calculated using the positive samples only, thus giving a skewed GMEPG result for the KK. Because the FEA-SD is more sensitive than the KK, it detects the high and low intensity infections, giving a more accurate assessment of estimation of current infection intensity.

The bovine contamination index (BCI) indicated that, on average, each carabao released more than 40,000 *S. japonicum* eggs into the environment daily, noting that the BCI was calculated conservatively using the lower end of the published daily fecal output rate for water buffaloes [8]. In comparison, Chinese water buffaloes have been shown to excrete ∼120,588 eggs per day (28.7 million *S. japonicum* eggs across 238 infected bovines) [9], [10]. In the Philippines, the habitats for the *Oncomelania* snails comprise streams, springs, canals, swampy areas, grass lands and rice fields, where carabao, other bovines and farmers work [17]. In the barangays, we observed that carabao were tethered with long ropes and were allowed to graze in communal areas, in or near rice paddy fields, and often grazed in or near water sources, particularly rivers. During the wet season (June–October for Manila and Northern Philippines; December–March for eastern Visayas and Mindanao) in the Philippines, bovines are either allowed to continue grazing in the rice fields or they are fed cut grass [33].

### Concluding comments

Based on the results obtained in this survey, we would recommend using either qPCR or FEA-SD in future surveys of large animals, such as bovines, for *S. japonicum*, as these techniques have a much higher sensitivity than the MHT or the KK and should thus provide much better estimates of prevalence. An additional benefit of the FEA-SD is that it can be performed in the field. While the procedure requires the use of a centrifuge, this equipment is available in many regional laboratories in the Philippines, whereas thermocyclers for PCR are unlikely to be readily available. The FEA-SD technique is also considerably less expensive per sample at 68 cents per test, compared with $8.8 ($US) for qPCR when calculated based on the costs of the reagents and consumable materials required.

The overall prevalence of *S. japonicum* infection in carabao was high, suggesting that these animals play a greater role in transmission and human infection in the Philippines than previously appreciated. As shown in China, the treatment of bovines with praziquantel [9], [10], or their removal and replacement with motorized tractors [34], as part of a comprehensive control strategy based on interventions to reduce the rate of transmission of *S. japonicum* infection to snails, can be highly effective, resulting in a reduction in transmission and human infection.

The detection of eggs in the stool by microscopy is still the standard for diagnosis. Despite the sensitivity of molecular and immunological techniques, eggs still need to be visualized for validation of these techniques. In the Leyte study [19], which estimated 51% prevalence of *S. japonicum* in bovines, the qPCR was not validated against egg counts. Indeed, eggs were found in <4% of samples using microscopic-based methods. In contrast, we employed the qPCR and the newly developed FEA-SD tool for more precise quantification of *S. japonicum* eggs in carabao feces, and by extensive microscopic examination of human fecal samples by two independent microscopists for the presence of eggs.

Recent studies in the Philippines have reported a low overall prevalence of schistosomiasis in previously classified endemic provinces [35], [36]. However, these surveys included non-endemic barangays, bringing down the overall prevalence for a province. Schistosomiasis is a focal disease and geographically close barangays can have varying infection status [37]. In this study, we found an unprecedentedly high prevalence of *S. japonicum* infection in humans, as well as in bovines This finding may be due to the insensitivity of the diagnostic tests used in previous studies and the low infection intensities, particularly in humans [5], [26], [35], [36], [38]. Despite the high prevalence recorded in humans, their precise involvement in the transmission of *S. japonicum* in the Philippines remains to be fully established. Additional surveys in Samar and other endemic areas of the Philippines should now be undertaken to determine the schistosomiasis-endemic status, in terms of human and animal infection prevalence and intensity. Nevertheless, the high *S. japonicum* BCI and prevalence data that we report for carabao suggest that, in the Philippines, they act, as in China [11]–[15], as important reservoir hosts. As such, future integrated control, including interventions targeting bovines, would likely reduce human incidence there.
